# Analysis of Mycorrhization Trends and Undesired Fungi Species in Three- and Six-Year-Old *Tuber aestivum* Plantations in Hungary

**DOI:** 10.3390/jof10100696

**Published:** 2024-10-07

**Authors:** Akale Assamere Habtemariam, Péter Cseh, Balázs Péter, Ádám Heller, Peter Pitlik, Sára Brandt, Péter László, Zoltán Bratek

**Affiliations:** 1 Department of Plant Physiology and Molecular Plant Biology, Eötvös Loránd University (ELTE), 1/C Pázmány Péter Sétány, 1117 Budapest, Hungary; petcseh01@gmail.com (P.C.); peterbalazs02@gmail.com (B.P.); helleradam03@gmail.com (Á.H.); peterpitlik@gmail.com (P.P.); bbavsz@gmail.com (S.B.); bratek.zoltan@ttk.elte.hu (Z.B.); 2 Department of Biology, Mekdela Amba University, South Wollo P.O. Box 32, Ethiopia; 3 Institute of Soil Sciences, HUN-REN Centre for Agricultural Research, 1022 Budapest, Hungary; laszlo.peter@atk.hun-ren.hu

**Keywords:** ectomycorrhizas, host plant affinity, soil pH, summer truffle

## Abstract

*Tuber aestivum* is a key truffle species with significant ecological and economic value. Despite its importance, plantation success can be influenced by soil pH, host plants, and undesired fungi. This study examines how soil pH and host plants influence mycorrhization trends in *T. aestivum* plantations across six plant species in eight Hungarian settlements, using root sampling and DNA analysis to assess plantations at three and six years of age. *Tuber aestivum* achieved over 30% mycorrhization, with *Carpinus betulus* showing the highest levels. DNA analysis identified eight undesired mycorrhizal fungi, with *Suillus* spp. (42.9%) and *Scleroderma* spp. (31.4%) being the most prevalent. The study found that *T. aestivum* preferred a soil pH of around 7.6, while undesired fungi thrived in slightly acidic conditions. Additionally, soil pH significantly and positively influenced *T. aestivum* mycorrhization; however, factors such as plantation age also contributed to mycorrhization trends. While mycorrhization by undesired fungi decreased with higher soil pH, it increased as plantations matured from three to six years. These findings highlight the need for the effective management of soil pH and the control of undesired fungi to optimize *T. aestivum* mycorrhization, emphasizing the importance of targeted strategies and further research for sustainable truffle cultivation.

## 1. Introduction

The summer truffle (*Tuber aestivum* Vittad.) is one of the most widely distributed truffles across Europe, found from Sweden to Spain and Morocco, and from the United Kingdom to Russia, with notable prevalence in Central Europe [1,2,3]. This truffle is naturally found in 26 of the 27 European Union member states, excluding Finland [4]. However, a report showed that cultivation also began in Finland between 2006 and 2008, with the first truffles found in 2012, proving successful growth in the boreal climate [5]. Historically, *T. aestivum* has been highly valued in cuisine due to its unique flavor and aroma, which have led to its increased popularity and high demand in culinary applications [6,7,8]. In Central Europe, particularly in the Jászság region of Hungary, *T. aestivum* has become a popular and economically significant truffle species due to its adaptability to various conditions [1,9,10,11,12]. Despite its adaptability, recent studies have reported a decline in natural truffle production due to various environmental factors [13,14].

Most truffles thrive best in calcareous soils with a pH between 7 and 8, but some species, such as *Tuber borchii*, can tolerate slightly acidic conditions [15,16]. Additionally, global climate change causes significant risks to truffle distribution and productivity [17,18,19]. In response to these environmental challenges, there has been increasing interest in establishing truffle plantations as a sustainable strategy to address production declines and meet the rising demand. In Hungary, this has been supported by initiatives such as the introduction of French seedlings in the 1990s and collaborations between French INRA and Eötvös Loránd University (ELTE), which have helped address these challenges and meet the increasing demand for *T. aestivum* [20,21].

In recent years, the renaissance of truffle plantations in Hungary has seen significant contributions from organizations such as Robin Nurseries, Agri-Truffle Ltd. (Saint-Maixant, France), and Pannon Szarvasgomba Ltd. (Budapest, Hungary), along with the help of the First Hungarian Truffle Society, which promotes sustainable practices in truffle farming [19]. *Tuber aestivum* plantations are now among the most widespread and economically important in Hungary [1,22]. However, a common issue in these plantations is the presence of undesired ectomycorrhizal fungi (they are important fungi but are not prominently desired in our plantations), which pose a challenge to the maintenance of healthy and productive truffle plantations. To understand and address these issues in the studied plantations, the study focused on examining how mycorrhizal colonization changes over time in three- and six-year-old truffle plantations, identified undesired fungi, and determined optimal host plants and soil pH for *T. aestivum*. The results will provide insights for refining cultivation practices, managing fungal competition, and ultimately enhancing the success of truffle plantations in Hungary. However, further research will be necessary to refine these strategies and explore additional factors influencing truffle production.

## 2. Materials and Methods

### 2.1. Description of Plantations and the Host Plant Species

In the Carpatho-Pannon region of Hungary, a total of 11 experimental plantations were established between 2004 and 2006. The project was a collaborative effort involving the French National Institute for Agricultural Research (INRA) and Eötvös Loránd University (ELTE). The primary aim of the project was establishing extensive plantations in Hungary using certified *T. aestivum* mycorrhized seedlings imported from Robin Nurseries, in Saint Laurent du Cros, France. The selected plantation sites were distributed across various locations, including Fiad, Gyúró, Hőgyész (with sites designated as 3, 4.1, 4.2, and 5), Jászszentandrás, Kiskunfélegyháza (sites 1 and 2), Szilvásvárad, and Buják. Unfortunately, the Buják plantation was destroyed by wild boars. In 2008, additional plantations were established in Gyöngyöspata and Solt, using seedlings provided by Pannon Szarvasgomba Ltd. and Agri-Truffle Ltd., respectively, as documented [4,20,21]. Plantations found in Solt and Gyöngyöspata received irrigation, while Fiad, Gyúró, Kiskunfélegyháza II, and Szilvásvárad benefitted from their proximity to the river. Topographically, Fiad, Gyúró, and Szilvásvárad are in valleys, Kiskunfélegyháza I is on a dry hill, Solt and Kiskunfélegyháza II are on flat plains, and Gyöngyöspata is on a northern slope.

**Figure 1:**
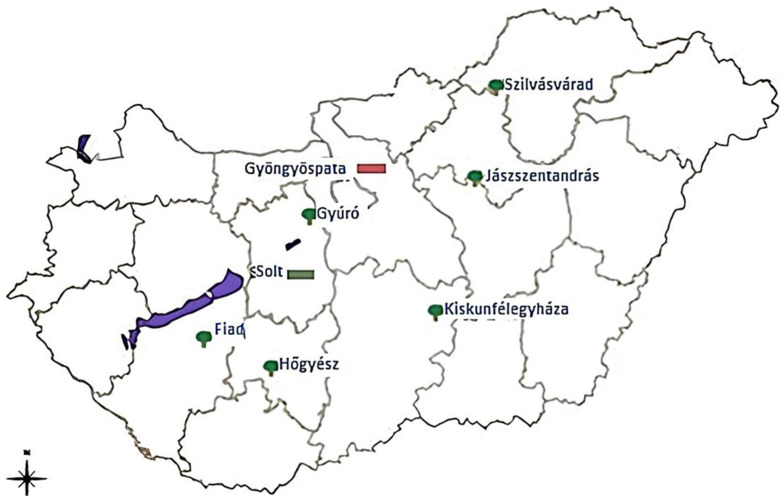
The distribution and locations of eight truffle plantation settlements across Hungary.

This study focused on 12 purposefully selected truffle plantations from eight locations in Hungary (Figure 1), all established between 2004 and 2008. The data for these plantations were obtained from our truffle plantation database, excluding the Buják plantation, which was destroyed by wild boars [23]. These plantations were studied at two key developmental stages: three years and six years after establishment. In all plantations, seedlings were planted with a spacing of 3 to 4 m between each plant, and each plant received a standardized quantity of *T. aestivum* spore inoculum.

The host plants were randomly selected, with the species and quantity of individual plants as follows: *Carpinus betulus* (188 specimens), *Corylus avellana* (189 specimens), *Pinus nigra* (135 specimens), *Quercus petraea* (49 specimens), *Quercus robur* (127 specimens), and *Quercus pubescens* (81 specimens) (see Supplementary Table S1 for detailed data). The second round of examination was conducted when the plantations were six years old, with a detailed analysis conducted on randomly re-selected plants, including 56 *C. betulus*, 31 *C. avellana*, 15 *Q. pubescens*, and 72 *Q. robur* plants. This re-examination aimed to assess the trends and effectiveness of truffle symbiosis in the plantations.

### 2.2. Soil Sampling and pH Measurement

In these plantations, numerous soil analyses were performed. However, this study focused exclusively on soil samples that contained comprehensive data and were collected from the root rhizosphere, ensuring that the information for evaluating the plantations was both ideal and concise for evaluating the plantations. Therefore, a total of 174 soil samples were systematically collected from the same locations as the root samples, specifically from the rhizosphere of the host plants, to maintain consistency in environmental conditions. The samples were taken at uniform depths and distances from the stems, and then carefully labeled and stored at 4 °C for subsequent analysis. The WTW InoLab pH Level 2 instrument (WTW made in Weinheim, Germany) was used to measure soil pH, with quality control ensured by calibrating the instrument according to the manufacturer’s instructions using four certified technical buffer solutions with pH values of 2, 4.01, 7, and 10 (models TEP 2, 4, 7, and 10, respectively).

Soil chemical analysis was conducted (Table 1) using the standard methods outlined [24], ensuring consistent and reliable results across samples. The pH of the soil was measured in water following the protocol established [25], although soil pH data for the Hőgyész 3 plantation was unavailable at the time of analysis. However, it is remarkable that the four plantations at the Hőgyész site (3, 4.1, 4.2, and 5) shared similar environmental conditions and nearly the same soil pH levels. This similarity suggests that the soil is consistent across these sites, allowing us to confidently use data from the other plantations to estimate conditions at Hőgyész 3.

### 2.3. Root Sampling Techniques, Mycorrhizal Assessment Methods and Analysis

Mycorrhizal examination was carried out at two distinct stages of plantation development. The initial assessments were conducted on a total of 769 plant roots when the plants in the plantations were three years old. This early evaluation aimed to assess the establishment and initial colonization of mycorrhizal fungi in the root systems. A follow-up examination was conducted at the six-year stage, focusing on 174 of the previously randomly selected plants to evaluate the dynamics of mycorrhizal colonization over time.

Roots were collected from the northeast edges of the root systems, within a 3–50 cm radius from the stem collar. For each plant, at least 300 root tips were examined microscopically, with each rootlet measuring about 5–10 cm in length [26,27]. Mycorrhizal colonization was quantified as the percentage of root tips colonized by *T. aestivum* relative to the total number of root tips examined [28].

### 2.4. Morphological and Molecular Studies and Phylogenetic Analysis

The morphological structure of the mycorrhizal was examined using a Nikon SMZ-U stereomicroscope, which allowed for detailed observation of the external features, while the mantle outer layers were examined using a Nikon Optiphot-2 research microscope (Nikon Co., Tokyo, Japan). *Tuber aestivum* mycorrhization was identified by Dr. Zoltán Bratek, who is an expert in truffle plantations in Hungary. For detailed identification, standard descriptions of *Tuber* species as outlined [29] were used. These descriptions provided comprehensive criteria for accurately distinguishing *T. aestivum* from other truffle species.

For molecular analysis, DNA was extracted from an expected non-targeted ectomycorrhizal fungus using the DNeasy Plant Mini Kit (Qiagen). Polymerase Chain Reaction (PCR) was performed using the ITS1-F/ITS4 primer pair [30,31], with amplification carried out on Bioer Little Genius TC-25/H and Techne TC-312 thermal cyclers. The PCR products were subsequently verified, purified, and sequenced to confirm their identity. Phylogenetic analysis was conducted using established procedures and software, following methodologies described in previous studies [19,32].

### 2.5. Statistical Analysis

Statistical analysis of ectomycorrhizal colonization was performed using XLSTAT (version 2022.2.1) and JASP (version 0.17.3). A simple Analysis of Variance (ANOVA) was used to determine significant differences among the means of colonization levels, with significance tested at *p* < 0.05. To further investigate specific differences in mycorrhization levels across plant species, Games–Howell post hoc comparisons and Tukey’s test were applied.

## 3. Results

### 3.1. Tuber Aestivum Plantations and Overview of Ectomycorrhizal Colonization

**Figure 2:**
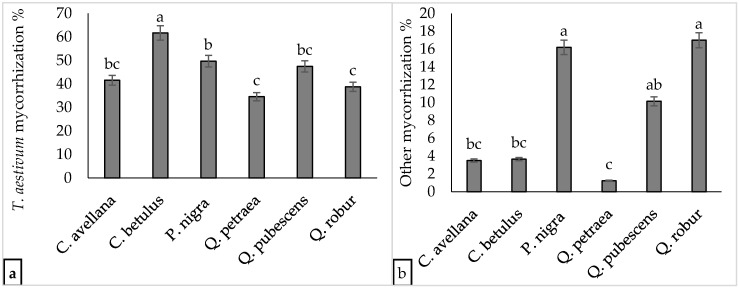
The levels of (**a**) *Tuber aestivum* mycorrhization and (**b**) contamination in six plant species from three-year-old plantations. Means that do not share the same letter are statistically significantly different from each other.

In this study, all six plant species examined exhibited mean mycorrhizal colonization levels exceeding 30%. The highest colonization level (61.67%) was observed in *C. betulus*, which was found in 10 plantations, with the highest and the leading mycorrhization levels recorded in 7 of them. This indicates significant ecological success and highlights its potential to thrive in various plantation environments. Following *C. betulus*, *P. nigra* showed higher mycorrhizal colonization than *Q. pubescens*, *C. avellana*, *Q. robur*, and *Q. petraea*. Undesired ectomycorrhizas were most frequently found in *Q. robur* (16.99%), followed by *P. nigra* (16.19%) and *Q. pubescens* (10.15%). In contrast, *Q. petraea*, *C. avellana*, and *C. betulus* had lower undesired mycorrhization levels, each below 5% (see Figure 2 and Supplementary Table S1).

**Figure 3:**
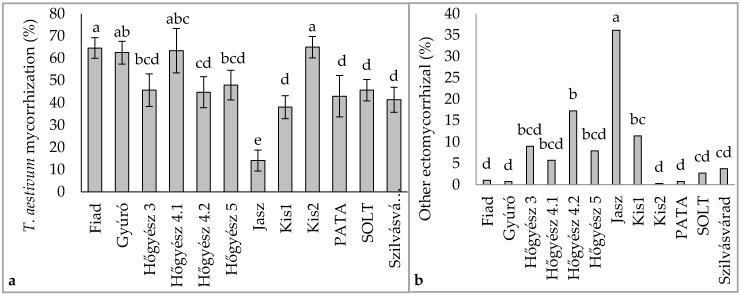
The levels of (**a**) *T. aestivum* and (**b**) undesired mycorrhizations across 12 plantations.

In the studied plantations, mycorrhizal colonization levels generally exceeded 30%, except for the Jászszentandrás plantation (see Supplementary Table S2). In the Kis2 plantation, targeted mycorrhizal colonization levels were the highest among all sites, with relatively low levels of undesired fungal species colonization. However, in the Jászszentandrás plantation, we observed the highest level of undesired ectomycorrhizal colonization (36.17%). In contrast, plantations at Fiad, Gyúró, Kis2, PATA, SOLT, and Szilvásvárad maintained undesired mycorrhizal levels below 5%, which are considered tolerable (refer to Figure 3 and Supplementary Table S2).

### 3.2. Effect of Plantation Age and Soil pH on Mycorrhizal Colonization Levels

**Figure 4:**
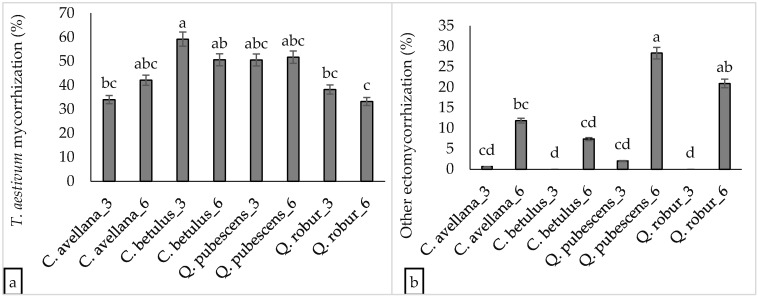
The levels of (**a**) *Tuber aestivum* mycorrhization and (**b**) undesired fungi across four plant species in three- and six-year-old plantations. Means that do not share a letter are significantly different.

**Figure 5:**
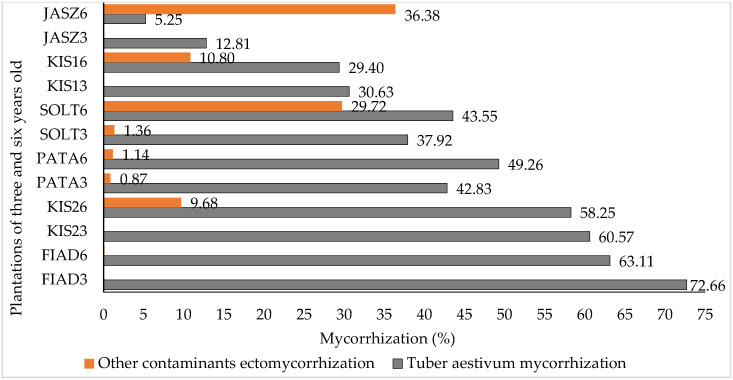
The levels of *T. aestivum* and undesired mycorrhization in different three- and six-year-old plantations.

In these sub-topics, the result presented here are based on 174 plants that were analyzed at both three and six years of age. Our results revealed that the highest mycorrhization of *T. aestivum* was reported in three-year-old *C. betulus* (59.17%). Significant variability in *T. aestivum* mycorrhizal levels was observed among some three-year-old plant species and among certain six-year-old plant species (see Figure 4 and Supplementary Table S3). Among the three-year-old plantations, the highest level of undesired mycorrhization was reported from *Q. pubescens* roots (2.03%). However, this level of undesired mycorrhization increased significantly to 28.33% by the age of six (see Supplementary Tables S4 and S5). However, relatively low mycorrhization levels of *T. aestivum* (12.81%) were observed in the three-year-old JASZ plantation. This colonization level declined by nearly half by the age of six (see Figure 5). In the three-year-old plantations, levels of undesired mycorrhization were ≤1.35% in the FIAD_3, JASZ_3, KIS1_3, and KIS2_3 plants. In comparison, the three-year-old SOLT_3 plantation exhibited the highest undesired ectomycorrhizal colonization level (1.35%) among similar-aged plantations. However, after three years, undesired mycorrhization levels increased significantly, reaching 9.68% in KIS2_6, 10.8% in KIS1_6, 29.72% in SOLT_6, and 36.38% in JASZ_6 plantations.

**Figure 6:**
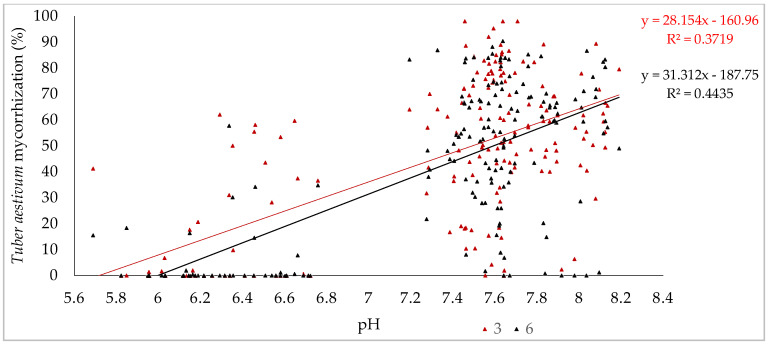
A scatter plot combined with a linear regression model illustrating the relationship between *T. aestivum* mycorrhization levels and soil pH in three- and six-year-old plantations.

**Figure 7:**
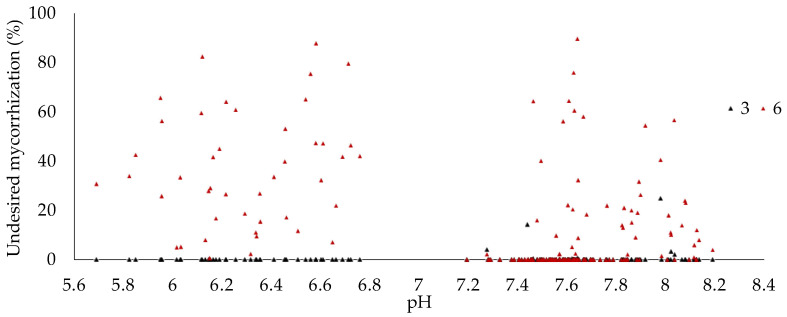
Scatter plots showing the mycorrhization levels of undesired fungi in *T. aestivum* plantations and their relationship with soil pH in three- and six-year-old plantations.

An important result of this study is the contribution of soil pH to the change in mycorrhization trends. The relationship between soil pH and *T. aestivum* mycorrhization in three- and six-year-old plants shows moderate significance, with correlation coefficients of r = 0.609 and r = 0.665, respectively. However, soil pH alone accounts for only 37.2% and 44.3% of the changes in mycorrhization levels, meaning that other factors also affect truffle mycorrhization (Figure 6). Additionally, the scatter plot revealed that many samples with higher mycorrhization levels were clustered near a pH of 7.6, as shown in Figure 6 and detailed in Table 2. In contrast, the study found that slightly acidic soils had some association with mycorrhization by undesired fungi, but this was not statistically significant; instead, plantation age (from three to six) was a more influential factor, as shown in Figure 7.

These results were supported by both the Pearson correlation matrix and simple ANOVA modeling. The test of the Pearson correlation matrix revealed no significant correlation between plantation age (from three to six years) and *T. aestivum* mycorrhization (r = −0.061, *p* = 0.257). However, a significant positive correlation exists between plantation age and undesired mycorrhizations (r = 0.431, *p* < 0.0001). Additionally, *T. aestivum* mycorrhization and undesired mycorrhizations show a significant negative correlation (r = −0.426, *p* < 0.0001). A strong positive correlation is found between *T. aestivum* mycorrhization and pH (r = 0.637, *p* < 0.0001). Finally, undesired mycorrhizations show a weak negative correlation with pH (r = −0.276, *p* < 0.0001), as shown in Table 3.

### 3.3. Undesired Ectomycorrhizal Fungi and Fungal Fruit Bodies in T. aestivum Plantations

**Figure 8:**
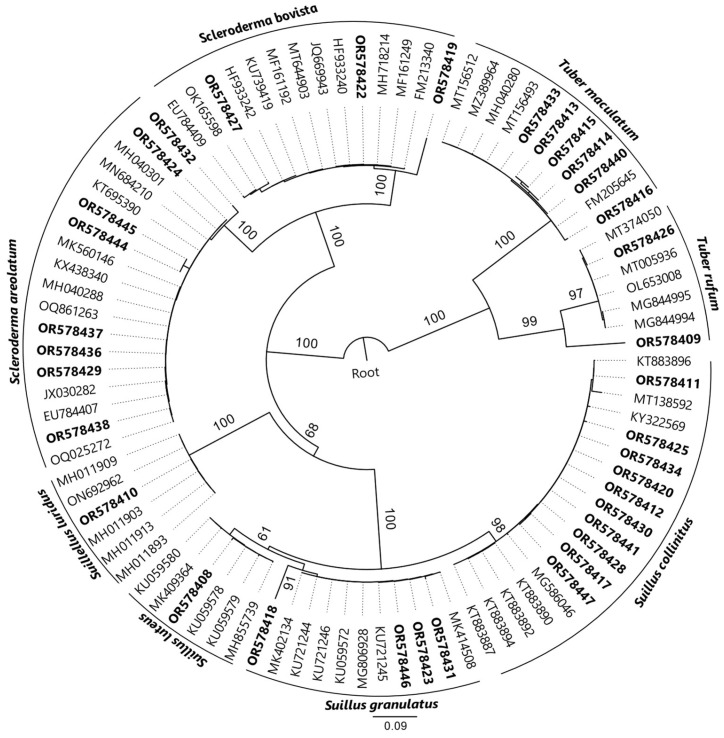
Rooted maximum likelihood phylogenetic tree illustrating evolutionary relationships among ectomycorrhizal fungal lineages identified in this study using IQ-TREE. Significant bootstrap support (>81) is as indicated above branches and bold letters denote sequences in this study.

Molecular analysis revealed the presence of eight distinct ectomycorrhizal fungal species in the *T. aestivum* plantations (see Figure 8 and Supplementary Table S6). The identified species included two white truffle species (*Tuber maculatum*, *Tuber rufum*), three *Suillus* species (*Suillus granulatus*, *Suillus luteus*, and *Suillus collinitus*), two *Scleroderma* species (*Scleroderma areolatum*, *Scleroderma bovista*), and *Suillellus luridus* (Figure 8, Supplementary Table S6). The genus *Suillus* was the most widespread among the identified ectomycorrhizas, constituting 42.9% of the total fungal community. Within this genus, *S. collinitus* was particularly dominant, accounting for 28.6% of the total samples. The species from the *Scleroderma* genus comprised 31.4% of the identified ectomycorrhizas, whereas *S. luteus* and *S. luridus* were detected in only one plant each, indicating their relatively rare occurrence in the studied plantations.

The study identified at least two distinct ectomycorrhizal fungi associated with the roots of each plant species analyzed. The most frequently reported fungi were *S. collinitus* (obtained from *P. nigra*), *S. granulatus* (also from *P. nigra*), *S. areolatum* (from *Quercus* spp.), *S. bovista* (from *Quercus* spp.), and *T. maculatum* (from *C. betulus*). Among these, *S. collinitus* was particularly dominant in *P. nigra* roots, while *S. granulatus*, *S. areolatum*, and *T. maculatum* were also present but less prevalent (see supplementary Figure S1).

**Figure 9:**
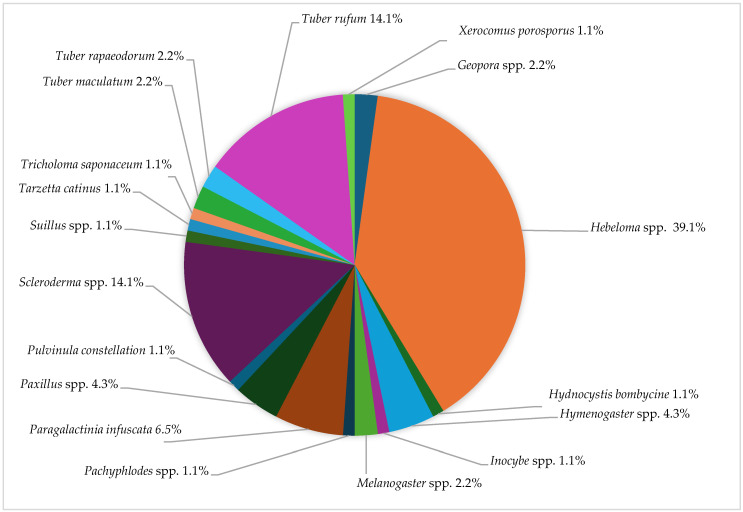
The frequency of morphologically identified ectomycorrhizal fruiting bodies in plantations.

In this study, a total of 18 different genera of undesired fruit bodies of the fungi were identified across the plantations, with these fruiting bodies found at distances ranging from 10 cm to 1 m from the host plants. Among these, *Hebeloma* spp. was the most frequently observed, comprising 39.1% of the total fruiting bodies harvested. Following *Hebeloma* spp., *Scleroderma* spp. and *T. rufum* were the next most prevalent, each representing 14.1% of the fruiting bodies. These findings are detailed in Figure 9 and Supplementary Table S7, which provide a comprehensive overview of the distribution and occurrence of these fungal genera within the plantations.

## 4. Discussion

Our results indicate that the extent of mycorrhizal colonization is influenced by the type of plant species, plantation age, and soil pH. Our findings are in line with previous research by [5] from Finland, which reported *T. aestivum* mycorrhization across all examined sites. The transition from controlled greenhouse conditions to natural field settings led to a notable increase in the colonization of root tips by *T. aestivum* [33]. Specifically, their study found that the colonization rate increased from 40% after six months in a greenhouse to 75% after one year in the field. Concurrently, they also found that contamination levels decreased from 54% to 25%. The study observed a significant increase in the colonization of root tips by *T. aestivum* over time. Our results indicate an absence of a strong correlation between plantation age and *T. aestivum* mycorrhization, suggesting that factors other than age may play a more critical role in determining mycorrhization levels. Conversely, we observed a significant positive correlation between plantation age and undesired mycorrhizations (r = 0.431, *p* < 0.0001), indicating that undesired mycorrhizations tend to increase over time. Furthermore, our findings showed that higher levels of *T. aestivum* mycorrhization were associated with fewer undesired mycorrhizations, while lower pH levels may promote the occurrence of undesired mycorrhizations. The results reported that an increase in mycorrhization levels of undesired fungi, accompanied by a more than 50% decline in *T. aestivum* mycorrhization between 2007 and 2010 [5]. This trend may be attributed to the presence of other undesired fungi either already present on the truffle farm or introduced from external sources.

Our data reveal a fivefold increase in undesired fungal colonization over three years, with most plantations exceeding 5% mycorrhization, except for the FIAD_6 plantation, which remained close to 1%. The colonization levels of undesired fungi in the plantations do not meet the European regulations for truffle cultivation [34]. Among the studied plantations, the highest mycorrhization of undesired fungi was observed in Jászszentandrás (JASZ), where slightly acidic soils were present. The proximity of older *Populus* trees in this plantation may have contributed to the spread of undesired fungal species. In terms of host plant compatibility, *C. betulus* has proven to be one of the most effective host plants for *T. aestivum* mycorrhization. Similarly, *P. nigra* also supports *T. aestivum* colonization effectively, although it requires careful management due to the presence of host-specific undesired fungi such as *Suillus* spp. Supporting our findings, the study identified *C. betulus* as the optimal host plant for *T. aestivum* in French truffle orchards, with *C. avellana*, *P. nigra*, *Q. pubescens*, and *Tilia cordata* also being favorable [35]. Additionally, study confirmed that *P. nigra* is a superior host plant compared to *Q. pubescens* and *Q. cerris* in Italian truffle cultivation [36]. These studies emphasize the importance of selecting appropriate host plants to optimize truffle production while managing contamination risks.

Another important finding of this study is that soil pH contributes a significant role in the distribution of mycorrhization. The mycorrhizal levels of *T. aestivum* increased within a defined pH range of 5.8 to 7.6, which aligns with previous study who reported a positive correlation between mycorrhization and soil pH up to 7.51 [37]. Meanwhile several studies emphasized an optimal pH range of 7–8 for truffle species [5,15,16,38,39]. Our study found mycorrhization occurring over a relatively wider pH range of 5.84–8.19, which is different from the narrower range of 7.13–7.89 reported by Bragato et al. (2021) [12] for *T. aestivum* in the Jászság region. However, our study also found the highest desired colonization levels around pH 7.6. Overall, these results confirm that soil pH significantly affects mycorrhizal colonization of *T. aestivum*.

The finding of molecular analysis revealed that *Suillus* spp., *Scleroderma* spp., and small white truffles were the most frequently found undesired mycorrhizal fungi. The significant presence of *Suillus* spp. and *Scleroderma* spp. may indicate their adaptability or competitive advantage in the local soil conditions, potentially influencing the establishment and growth of *T. aestivum*.

According to previous study, *Scleroderma* spp., *Pisolithus* spp., and *Hebeloma* spp. are among the most common undesired ectomycorrhizal fungi in truffle plantations [40]. In contrast, our study found *Hebeloma* spp., *Scleroderma* spp., and *Tuber rufum* as the most frequently encountered fruit bodies, with *S. collinitus* being the most dominant mycorrhizal colonizer in plant roots. The high competitiveness of *Hebeloma* spp. has been well documented [41] and poses a significant threat to truffle production. Additionally, the host-specific association between *S. collinitus* and *P. nigra* could potentially impact *T. aestivum* mycorrhization. Therefore, the overall results of our study emphasize the critical need for active management to prevent an increase in mycorrhization levels of undesired fungi, which poses a risk for replacing the target truffle species, *T. aestivum*. The study indicates that without such management, these fungi can quickly become dominant, threatening the desired truffle species.

## 5. Conclusions

The global expansion of truffle plantations has been remarkable, particularly in the 21st century, with a notable increase in large-scale establishments. Successful truffle cultivation depends on several factors, with soil pH being crucial. This study highlights the successful establishment of *Tuber aestivum* mycorrhization in various plantations. Although some plantations, such as Kis2, maintained low undesired colonization levels, Jászszentandrás exhibited a high undesired mycorrhization. *Tuber aestivum* mycorrhization is most effective at a pH of around 7.6, where undesired fungi are less common. Plantations outside this pH range are more likely to suffer from undesired fungi. Additionally, the choosing of host plant species significantly impacts the effectiveness of mycorrhizal relationships and overall plantation success. Age and soil pH are also influential factors; specifically, older plantations may be vulnerable to undesired mycorrhization. Therefore, the effective management of soil pH and the careful selection of host plants are essential for successful truffle cultivation. Moreover, once undesired fungi have been detected in a plantation, inadequate monitoring and management can lead to a rise in aggressive and species-specific undesired fungi as the plantation ages. Therefore, regular monitoring and timely intervention are crucial for addressing these issues and ensuring the long-term success of truffle plantations.

## Figures and Tables

**Table 1:** The physical and chemical properties of the soil from various *T. aestivum* plantations.

Plantation	Soil Composition				Soil Physical Characteristics in the Plantation
	CaCO_3_ (m/m%)	Ca (mg/kg)	Salt	pH (H_2_O)	
Fiad	10.39	2.58	<0.02	7.8	Brown with light brown loam
Gyöngyöspata	1.6	1.15	<0.02	7.5	Clay
Gyúró	8.52	2.86	<0.02	7.68	Brown silt from 10 cm
Jászszentandrás	0	0.4	<0.02	6.7	Brown sand with humus
Hőgyész 4.1	4.35	1.73	<0.02	7.56	Dark brown humus layer, clay loam
Hőgyész 4.2	17.25	4.38	<0.02	7.66	-
Hőgyész 5	15.55	3.94	<0.02	7.77	Gray fine to light brown fine sand
Kiskunfélegyháza I	0.07	3.01	<0.02	8.27	Brown sand with humus
Kiskunfélegyháza II	0.24	4.98	<0.02	8.14	Brown sand with humus
Solt	6.87	2.82	<0.02	8	Brown sand with humus
Szilvásvárad	6.11	2.82	0.05	7.82	Brown, humus level silty clay

**Table 2:** A descriptive summary of the mycorrhization levels of *T. aestivum* and undesired fungi and soil pH values across six different plantations.

Plantation	No.	Mycorrhization (%)	Undesired Mycorrhization (%)	pH (H_2_O)	Host Plants
FIAD_3	45	72.7 ± 17.6	0.0	7.6 ± 0.10	*C. betulus* and *Q. robur*
FIAD_6	45	63.1 ± 18.6	0.1 ± 0.5		
JASZ_3	43	12.8 ± 21.2	0.0	6.3 ± 0.27	*C. betulus* and *Q. robur*
JASZ_6	43	5.3 ± 12.4	36.4 ± 23.2		
KIS1_3	8	30.6 ± 30.4	0.0	7.6 ± 0.21	*Q. robur* and *C. avellana*
KIS1_6	8	29.4 ± 27.6	10.8 ± 19.3		
KIS2_3	33	60.6 ± 13.6	0.0	7.9 ± 0.17	*Q. robur* and *C. betulus*
KIS2_6	33	58.3 ± 22.9	9.7 ± 12.5		
PATA_3	21	42.8 ± 18.8	0.9 ± 3.2	7.4 ± 0.19	*C. avellana*
PATA_6	21	49.3 ± 15.2	1.1 ± 4.8		
SOLT_3	24	37.9 ± 23.6	1.4 ± 5.1	7.7 ± 0.20	*C. avellana* and *Q. pubescens*
SOLT_6	24	43.6 ± 29.4	29.7 ± 27.8		

**Table 3:** The Pearson correlation matrix test between four variables: age of plantation, *T. aestivum* mycorrhization, undesired mycorrhizations, and soil pH. Values in bold have a significance level of alpha = 0.05.

Variables 1	Variables 2	Correlation Matrix (r)	*p*-Value
Age of plantations (from three to six years)	*Tuber aestivum* mycorrhization	−0.061	0.257
	Undesired mycorrhization	**0.431**	**<0.0001**
*Tuber aestivum* mycorrhization	Undesired mycorrhization	**−0.426**	**<0.0001**
	pH	**0.637**	**<0.0001**
Undesired mycorrhizations	pH	**−0.276**	**<0.0001**

## Data Availability

All data is available in the manuscript.

## Supplementary Materials

The following supporting information can be downloaded at: https://www.mdpi.com/article/10.3390/jof10100696/s1, Figure S1: The frequency of undesired ectomycorrhizal colonization across plant species; Table S1: The overall descriptive statistics of *T. aestivum* mycorrhization and other ectomycorrhizal colonization in different plant species at the age of three; Table S2: Overall descriptive statistics of *T. aestivum* mycorrhization and other ectomycorrhizal colonization in relation to plantations at the age of three; Table S3: The Games-Howell Post-hoc comparisons of the significant differences between different host plant species depending on *T. aestivum* mycorrhization and other undesired ectomycorrhizal levels; Table S4: Descriptive statistics of *Tuber aestivum* and other ectom ycorrhizal colonization levels in four different plant species at the ages of three and six; Table S5: The Games-Howell Post-hoc comparisons of different host plant species in three- and six-year-old plantations based on ectomycorrhizal levels; Table S6: Details on the similarity of ectomycorrhizal sequences to those obtained through BLAST analysis, the year of collection, and the host plants from which the samples were obtained across different plantations; Table S7: The frequency of the identified genus of ectomycorrhizal fruiting bodies in plantations.

## Abbreviations

**Original Name of the plantations**	**Abbreviations used in the article**
Kiskunfélegyháza I	KIS 1
Kiskunfélegyháza II	KIS 2
Gyöngyöspata	PATA
Jászszentandrás	JASZ
