# Supplementary material for: Analysis of Mycorrhization Trends and Undesired Fungi Species in Three- and Six-Year-Old Tuber aestivum Plantations in Hungary
Source: J Fungi (Basel). 2024 Oct 7;10(10):696. doi: 10.3390/jof10100696 (PMC11508518; doi:10.3390/jof10100696)
Supplement: Supplementary file 1 [file jof-10-00696-s001.zip › jof-3192304-supplementary.pdf]

Supplementary Materials

Supplementary Figure

Figure S1. The frequency of undesired ectomycorrhizal colonization across plant species

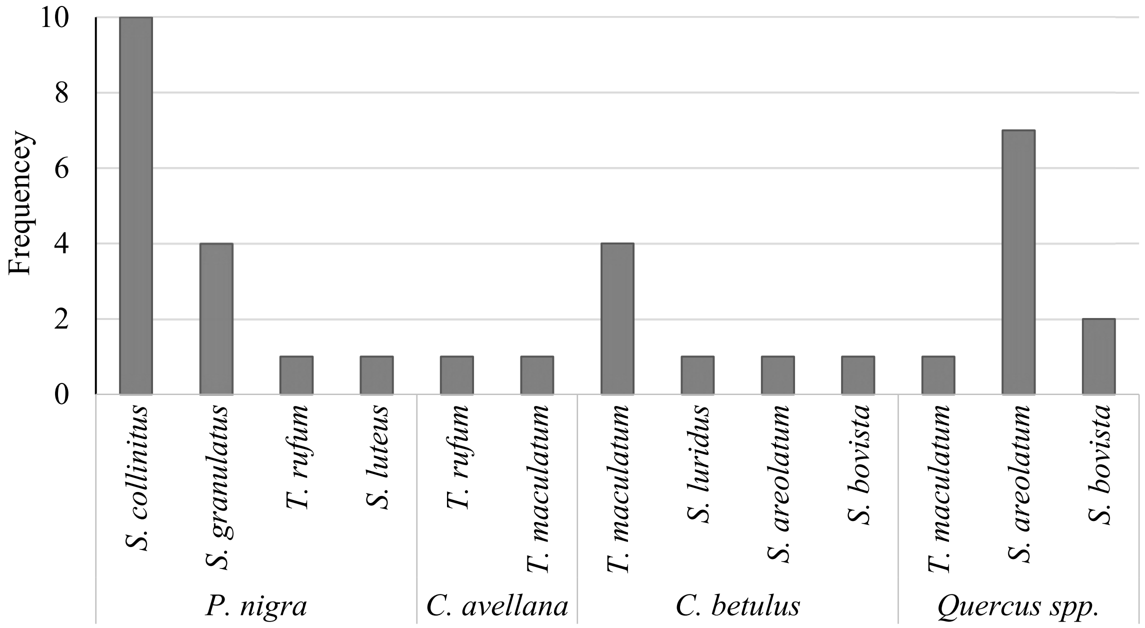

Supplementary Tables

Table S1. The overall descriptive statistics of *T. aestivum* mycorrhization and other ectomycorrhizal colonization in different plant species at the age of three.

Variable	Host plant species	N	Mean	SE Mean	StDev	Minimum	Maximum
Tuber aestivum mycorrhization (%)	*Carpinus betulus*	188	61.67	2.01	27.51	0.00	98.00
	Corylus avellana	189	41.54	1.90	26.16	0.00	88.30
	Pinus nigra	135	49.69	2.67	31.06	0.00	100.00
	Quercus petraea	49	34.56	4.37	30.56	0.00	92.00
	Quercus pubescens	81	47.46	2.64	23.73	0.00	84.78
	Quercus robur	127	38.76	2.58	29.11	0.00	94.90
Other ectomycorrhizal colonization (%)	*Carpinus betulus*	188	3.659	0.921	12.627	0.00	87.00
	Corylus avellana	189	3.499	0.930	12.790	0.00	78.00
	Pinus nigra	135	16.20	1.92	22.34	0.00	86.40
	Quercus petraea	49	1.235	0.854	5.978	0.00	39.60
	Quercus pubescens	81	10.15	2.58	23.26	0.00	92.00
	Quercus robur	127	16.99	2.69	30.33	0.00	100.00

Table S2. Overall descriptive statistics of *T. aestivum* mycorrhization and other ectomycorrhizal colonization in relation to plantations at the age of three.

Variable	Plantations	N	Mean	SE Mean	StDev	Minimum	Maximum
T. aestivum mycorrhization	Fiad	98	64.69	2.46	24.32	2.90	100.00
	Gyúró	79	62.58	2.65	23.60	0.00	97.60
	Hőgyész 3	39	45.71	4.67	29.18	0.00	92.00
	Hőgyész 4.1	21	63.47	4.38	20.09	22.20	88.30
	Hőgyész 4.2	43	44.78	3.43	22.48	0.30	88.30
	Hőgyész 5	47	48.00	3.54	24.30	0.00	77.00
	Jasz	94	14.10	2.20	21.33	0.00	72.08
	Kis1	78	38.08	3.72	32.83	0.00	100.00
	Kis2	90	65.03	1.90	18.05	0.00	93.10
	PATA	24	43.02	3.59	17.58	16.74	75.40
	SOLT	90	45.74	2.40	22.80	0.00	84.78
	Szilvásvárad	66	41.44	3.94	31.98	0.00	90.70
Other ectomycorrhizal %	Fiad	98	1.028	0.360	3.565	0.000	19.900
	Gyúró	79	0.730	0.341	3.034	0.000	21.200
	Hőgyész 3	39	9.02	3.60	22.51	0.00	87.00
	Hőgyész 4.1	21	5.70	3.22	14.74	0.00	49.00
	Hőgyész 4.2	43	17.28	3.41	22.35	0.00	80.00
	Hőgyész 5	47	7.93	2.69	18.45	0.00	70.50
	Jasz	94	36.18	3.26	31.58	0.00	100.00
	Kis1	78	11.45	2.64	23.30	0.00	100.00
	Kis2	90	0.287	0.287	2.720	0.000	25.800
	PATA	24	0.763	0.610	2.990	0.000	14.253
	SOLT	90	2.74	1.12	10.62	0.00	77.26
	Szilvásvárad	66	3.72	1.63	13.24	0.00	72.80

Table S3. The Games-Howell Post-hoc comparisons of the significant differences between different host plant species depending on *T. aestivum* mycorrhization and other undesired ectomycorrhizal levels.

Ectomycorrhizas	Games-Howell Post Hoc Comparisons between plant species						
	**Comparison**		**Mean Difference**	**SE**	**t**	**df**	**ptukey**
T. aestivum mycorrhization	*C. betulus*	*C. avellana*	20.133	2.766	7.28	373.844	< .001***
		P. nigra	11.985	3.342	3.586	266.87	0.005**
		Q. petraea	27.117	4.805	5.643	69.62	< .001***
		Q. pubescens	14.209	3.313	4.289	174.486	< .001***
		Q. robur	22.911	3.271	7.004	260.114	< .001***
	P. nigra	*Q. petraea*	15.132	5.119	2.956	86.37	0.045*
		Q. robur	10.927	3.717	2.939	259.997	0.041*
Other ectomycorrhizas	*C. betulus*	*P. nigra*	-12.538	2.132	-5.881	195.169	< .001***
		Q. robur	-13.334	2.845	-4.687	155.786	< .001***
	C. avellana	*P. nigra*	-12.697	2.136	-5.944	196.415	< .001***
		Q. robur	-13.494	2.848	-4.738	156.408	< .001***
	P. nigra	*Q. petraea*	14.961	2.104	7.111	173.262	< .001***
	Q. petraea	*Q. pubescens*	-8.911	2.722	-3.274	96.512	0.018*
		Q. robur	-15.758	2.824	-5.58	148.69	< .001***

Where * $p < .05$, ** $p < .01$, *** $p < .001$. Note. Results based on uncorrected means.

Table S4. Descriptive statistics of *Tuber aestivum* and other ectomycorrhizal colonization levels in four different plant species at the ages of three and six.

**Variable**	**tree**	**N**	**Mean**	**SE Mean**	**StDev**	**Minimum**	**Maximum**
T. aestivum mycorrhization %	*C. betulus*_3	56	59.17	4.29	32.09	0.00	98.00
	*C. betulus*_6	56	50.58	4.34	32.47	0.00	90.41
	*C. avellana*_3	31	34.01	3.88	21.59	0.00	75.40
	*C. avellana*_6	31	42.08	3.84	21.37	0.00	74.01
	*Q. pubescens*_3	15	50.50	4.76	18.44	6.37	82.27
	*Q. pubescens*_6	15	51.67	7.65	29.62	0.00	88.22
	Q. robur _3	72	38.18	3.44	29.21	0.00	84.94
	Q. robur _6	72	33.21	3.50	29.71	0.00	85.30
Other ectomycorrhizal	*C. betulus*_3	56	0.0	0.0	0.0	0.0	0.0
	*C. betulus*_6	56	7.40	1.67	12.51	0.00	53.06
	*C. avellana*_3	31	0.660	0.472	2.627	0.000	14.253
	*C. avellana*_6	31	11.83	4.18	23.28	0.00	89.64
	*Q. pubescens*_3	15	2.03	1.65	6.38	0.00	24.84
	*Q. pubescens*_6	15	28.33	6.83	26.45	0.00	75.91
	Q. robur _3	72	0.0	0.0	0.0	0.0	0.0
	Q. robur _6	72	20.91	2.97	25.19	0.00	87.86

Table S5. The Games-Howell Post-hoc comparisons of different host plant species in three- and six-year-old plantations based on ectomycorrhizal levels.

Ectomycorrhizas	Games-Howell Post Hoc Comparisons – Host plant species						
Tuber aestivum mycorrhization	Plant species		Mean Difference	SE	t	df	ptukey
	*C. betulus*_3	*C. avellana*_3	25.159	5.781	4.352	81.648	< .001***
		*Q. robur*_3	20.992	5.499	3.818	112.522	0.005**
		*Q. robur*_6	25.961	5.536	4.689	113.651	< .001***
	*C. betulus*_6	*Q. robur* _6	17.367	5.575	3.115	112.878	0.047*
Other ectomycorrhizas	*C. betulus*_3	*C. betulus*_6	-7.403	1.671	-4.43	55	0.001**
		*Q. pubescens*_6	-28.33	6.828	-4.149	14	0.016*
		Q. robur _6	-20.912	2.968	-7.045	71	< .001***
	*C. betulus*_6	*C. avellana*_3	6.743	1.737	3.883	63.379	0.006**
		Q. robur _3	7.403	1.671	4.43	55	0.001**
		Q. robur _6	-13.509	3.407	-3.965	109.004	0.003**
	*C. avellana*_3	*Q. robur* _6	-27.67	6.845	-4.043	14.134	0.02*
		Q. robur _6	-20.252	3.006	-6.738	74.52	< .001***
	*Q. pubescens*_3	*Q. pubescens*_6	-26.301	7.024	-3.744	15.625	0.03*
		Q. robur _6	-18.883	3.395	-5.562	82.004	< .001***
	*Q. pubescens*_6	*Q. robur* _3	28.33	6.828	4.149	14	0.016*
	Q. robur _3	*Q. robur* _6	-20.912	2.968	-7.045	71	< .001***

Table S6**.** Details on the similarity of ectomycorrhizal sequences to those obtained through BLAST analysis, the year of collection, and the host plants from which the samples were obtained across different plantations.

Year	Plantation	Host plant	Blasted species	GenBank code	Blast code	Query coverage	Percent identity
2007	Fiad	*P. nigra*	*S. collinitus*	OR578420	MT138592	100	100
2009	Fiad	*P. nigra*	*S. collinitus*	OR578447	KT883887	100	100
2008	Gyúró	*P. nigra*	*S. collinitus*	OR578428	MT138592	100	99.79
2009	Gyúró	*C. betulus*	*T. maculatum*	OR578433	MT156493	100	100
2008	Hőgyész 3	*C. avellana*	*T. rufum*	OR578409	FM205676	100	93.27
2008	Hőgyész 3	*C. betulus*	*T. maculatum*	OR578413	MT156512	100	99.64
2008	Hőgyész 3	*Quercus*spp.	*T. maculatum*	OR578416	OM265279	100	98.97
2008	Hőgyész 3	*Quercus* spp.	*S. areolatum*	OR578445	MN684210	100	99.21
2008	Hőgyész 4.1	*C. betulus*	*S. luridus*	OR578410	MH011893	100	100
2008	Hőgyész 4.2	*P. nigra*	*S. luteus*	OR578408	MK409364	100	99.08
2009	Hőgyész 4.2	*P. nigra*	*S. collinitus*	OR578417	KT883887	100	99.84
2008	Hőgyész 4.2	*P. nigra*	*S. collinitus*	OR578425	MH040301	100	100
2008	Hőgyész 4.2	*P. nigra*	*T. rufum*	OR578426	MT374050	100	100
2008	Hőgyész	*C. betulus*	*T. maculatum*	OR578414	MT156512	99	99.64
2008	Hőgyész 5	*C. avellana*	*T. maculatum*	OR578415	MT156493	99.68	100
2007	Jászszentandrás	*P. nigra*	*S. granulatus*	OR578418	MK414508	92	95.47
2007	Jászszentandrás	*Quercus* spp.	*S. bovista*	OR578419	MT644903	80	98.21
2009	Jászszentandrás	*P. nigra*	*S. granulatus*	OR578423	MK414508	100	100
2009	Jászszentandrás	*Quercus* spp.	*S. areolatum*	OR578424	MT138592	100	99.45
2009	Jászszentandrás	*C. betulus*	*S. bovista*	OR578427	EU784409	100	99.74
2009	Jászszentandrás	*Quercus* spp.	*S. areolatum*	OR578429	MN684210	100	100
2009	Jászszentandrás	*P. nigra*	*S. granulatus*	OR578431	MK414508	100	99.63
2009	Jászszentandrás	*Quercus* spp.	*S. areolatum*	OR578432	JX030282	100	99.49
2009	Jászszentandrás	*Quercus* spp.	*S. areolatum*	OR578436	MH040288	100	100
2009	Jászszentandrás	*Quercus* spp.	*S. areolatum*	OR578437	KX438340	100	100
2009	Jászszentandrás	*C. betulus*	*S. areolatum*	OR578438	OQ025273	100	100
2009	Jászszentandrás	*C. betulus*	*T. maculatum*	OR578440	MT156512	100	100
2009	Jászszentandrás	*Quercus* spp.	*S. areolatum*	OR578444	MN684210	100	99.38
2009	Jászszentandrás	*P. nigra*	*S. granulatus*	OR578446	MK402134	100	100
2008	Kiskunfélegyháza I	*P. nigra*	*S. collinitus*	OR578411	KT883896	100	100
2008	Kiskunfélegyháza I	*P. nigra*	*S. collinitus*	OR578412	MT138592	100	100
2008	Kiskunfélegyháza I	*Quercus* spp.	*S. bovista*	OR578422	MT644903	100	99.81
2009	Kiskunfélegyháza I	*P. nigra*	*S. collinitus*	OR578430	MT138592	100	100
2009	Kiskunfélegyháza I	*P. nigra*	*S. collinitus*	OR578434	MT138592	100	100
2009	Kiskunfélegyháza II	*P. nigra*	*S. collinitus*	OR578441	MT138592	100	100

Tabel S7. The frequency of the identified genus of ectomycorrhizal fruiting bodies in plantations.

Year	Genus	Location	Frequency	Determinator
2013	*Geopora* spp.	Solt	2	Mihály, István,
2014	*Hebeloma* spp.	Gyöngyöspata	1	Feri, Paca, Őze,
2012-2018	*Hebeloma* spp.	Solt	35	Bratek Z, Tóth
2006	*Hydnocystis bombycina*	Hőgyész	1	Annamária
2014	*Hymenogaster* spp.	Gyöngyöspata	3	
2007	*Hymenogaster* spp.	Hőgyész	1	
2015	*Inocybe* spp.	Kiskunfélegyháza	1	
2014	*Melanogaster* spp.	Gyöngyöspata	2	
2011	*Pachyphlodes* spp.	Kiskunéfegyháza	1	
2019	*Paragalactinia infuscata*	Solt	6	

2014	*Paxillus* spp.	Gyöngyöspata	1	
2017	*Paxillus* spp.	Solt	3	
2016	*Pulvinula constellatio*	Solt	1	
2016	*Scleroderma* sp.	Gyöngyöspata	1	
2011	*Scleroderma* sp.	Jászszentandrás	1	
2011	*Scleroderma* sp.	Kiskunfélegyháza	1	
2016	*Scleroderma* sp.	Solt	10	
2013	*Suillus* spp.	Jászszentandrás	1	
2015	*Tarzetta* catinus	Kiskunfélegyháza	1	
2018	*Tricholoma saponaceum*	Solt	1	
2005	*Tuber maculatum*	Hőgyész	2	
2007	*Tuber rapaeodorum*	Hőgyész	2	
2014-2016	*Tuber rufum*	Gyöngyöspata	7	
2005	*Tuber rufum*	Hőgyész	4	
2016	*Tuber rufum*	Szilvásvárad	2	
2013	*Xerocomus porosporus*	Solt	1	
